# Novel Sources of Bioactive Molecules: Gut Microbiome of Species Routinely Exposed to Microorganisms

**DOI:** 10.3390/vetsci9080380

**Published:** 2022-07-25

**Authors:** Ruqaiyyah Siddiqui, Morhanavallee Soopramanien, Ahmad M. Alharbi, Hasan Alfahemi, Naveed Ahmed Khan

**Affiliations:** 1 College of Arts and Sciences, American University of Sharjah, Sharjah 26666, United Arab Emirates; rsiddiqui@aus.edu; 2 Department of Clinical Sciences, College of Medicine, University of Sharjah, Sharjah 27272, United Arab Emirates; morhana@gmail.com; 3 Department of Clinical Laboratory Sciences, College of Applied Medical Sciences, Taif University, Taif 21944, Saudi Arabia; a.alharbii@tu.edu.sa; 4 Department of Medical Microbiology, Faculty of Medicine, Al-Baha University, Al-Baha 65799, Saudi Arabia; halfahmi@bu.edu.sa

**Keywords:** gut microbiota, animal, polluted environment, anti-infective, antibacterial activity, intestinal microflora, antibiotic

## Abstract

**Simple Summary:**

The majority of antibiotics available in the market are produced by bacteria isolated from soil. However, the low-hanging fruit has been picked; hence, there is a need to mine bacteria from unusual sources. With this in mind, it is important to note that animals and pests, such as cockroaches, snake, crocodiles, water monitor lizards, etc., come across pathogenic bacteria regularly, yet flourish in contaminated environments. These species must have developed methods to defend themselves against pathogens. Besides the immunity they may confer, bacteria associated with animals/pests may offer a potential source of novel antibacterial agents. This paper discusses the current knowledge of bacteria isolated from land and marine animals with antibacterial properties and proposes untapped sources for the isolation of bacteria to mine potentially novel antibiotic molecules.

**Abstract:**

The development of novel bioactive molecules is urgently needed, especially with increasing fatalities occurring due to infections by bacteria and escalating numbers of multiple-drug-resistant bacteria. Several lines of evidence show that the gut microbiome of cockroaches, snakes, crocodiles, water monitor lizards, and other species may possess molecules that are bioactive. As these animals are routinely exposed to a variety of microorganisms in their natural environments, it is likely that they have developed methods to counter these microbes, which may be a contributing factor in their persistence on the planet for millions of years. In addition to the immune system, the gut microbiota of a host may thwart colonization of the gastro-intestine by pathogenic and/or foreign microorganisms through two mechanisms: (i) production of molecules with antibacterial potential targeting foreign microorganisms, or (ii) production of molecules that trigger host immunity targeting foreign microorganisms that penetrate the host. Herein, we discuss and deliberate on the current literature examining antibacterial activities that stem from the gut bacteria of animals such as crocodiles, cockroaches, and water monitor lizards, amongst other interesting species, which likely encounter a plethora of microorganisms in their natural environments. The overall aim is to unveil a potential library of novel bioactive molecules for the benefit of human health and for utilization against infectious diseases.

## 1. Introduction

Infectious diseases remain one of the leading causes of mortality worldwide, with more than 14 million deaths resulting from bacterial infections per year [1,2]. Bacterial infections have been observed to be more severe with the increasing number of multiple-drug-resistant bacteria. Such bacteria are referred to as ‘ESKAPE’ organisms, which stands for *Enterococcus* spp., *Staphylococcus aureus*, *Klebsiella* spp., *Acinetobacter baumannii*, *Pseudomonas aeruginosa,* and *Enterobacter* spp. [3,4]; therefore, the development of novel antimicrobials is warranted. In previous years, natural resources have been explored in the search for naturally occurring molecules with antibacterial properties from plants and microorganisms [5,6]. Since the discovery of the antibiotic penicillin from *Penicillium* molds by Alexander Fleming in 1928, it is well known that microorganisms are known to produce antibiotic molecules as a defense mechanism against other microorganisms [6,7,8]. Thus, previous research has focused on isolating antibacterial molecules from fungi/bacteria living in the soil [9,10]. The expression of antimicrobial agents by bacteria is a defense mechanism whereby the stronger/most resistant strain can thwart sensitive bacteria, promoting its own biodiversity by dominating the ecosystem, and this also serves as communication signal [11,12]. Following the results from the Human Microbiome Project in 2012 [12], the understanding that our cells are outnumbered by bacteria has generated interest in the microorganisms that colonize our bodies, and led to a plethora of studies investigating the role of the microbiome in human health [13,14,15]. However, the gut microbiome of animals has received little attention. Herein, we provide an overview of several species whose gut microbiota have been evaluated for their anti-bacterial effects. This hypothesis-driven research is critical in our search for novel and much-needed antimicrobials and bioactive molecules. It is hoped that these novel bioactive molecules may also be utilized as probiotics for the benefit of human and animal health.

## 2. The Importance of the Gut Microbiome

First coined by Lederberg and McCray in 2001, “microbiome” refers to microorganisms inhabiting multicellular organisms, from animals to plants. The microbiome is made up of bacteria, archaea, protists, fungi, and viruses [15,16,17,18,19]. The gut contains 70–80% of the immune cells of its host, and is known to play a profound role in the overall health of the host. Intestinal epithelial cells interact with microbial products as well as luminal contents, and are able to secrete anti-microbial peptides with anti-inflammatory, bactericidal, and other beneficial properties [19]. Moreover, the microbiota may influence immune responses via the generation of metabolites from dietary constituents or the host itself [20]. A plethora of studies have examined the role of the microbiome in human health [21,22,23,24,25,26], however, studies on the gut microbiome of animals, which may be a rich source of novel bioactive molecules, are limited. Of note, the constitution of the host’s microbiota is dependent on the host’s genome, and reflects the co-evolution of the host and its microbes to achieve a balanced and mutually beneficial state. In fact, the relationship between a host and its microbiota is described as a symbiotic one, whereby both parties are known to benefit from one another [27]. The host provides the microorganisms with shelter and food, and the microbiota has been found to have key roles in nutrient metabolism, protection against pathogens and diseases, and immunomodulation.

The human gut microbiome has remained of interest for researchers over recent years, rather than that of animals [28]. However, animals possess both internal and external microbiota niches, which contain genes that outnumber those of the host by almost ten times [29]. While the external microbiota inhabits mostly the skin, the internal microbiota inhabits the oral cavity, the gastrointestinal tract, and the respiratory and urogenital tracts [18,30]. Furthermore, animal gut microbiota vary across species, as gastrointestinal tract morphology differs from animal to animal, thereby offering a broader range of bacteria to be considered. Moreover, since the external environment affects the constitution of host microbiota, it is noteworthy that animals that flourish in environments that may be damaging for humans, such as those laden with heavy metals where crocodiles reside and consume rotten meat [31]; or unhygienic environments, such as sewers or drains, where cockroaches thrive [32,33]; or the diet consumed by snakes and water monitor lizards, which may comprise live rodents and carcasses [34,35]. We hypothesize that these animals may possess various mechanisms to ward off disease, allowing them to thrive for years in these conditions [36,37,38,39,40,41,42].

**Figure 1:**
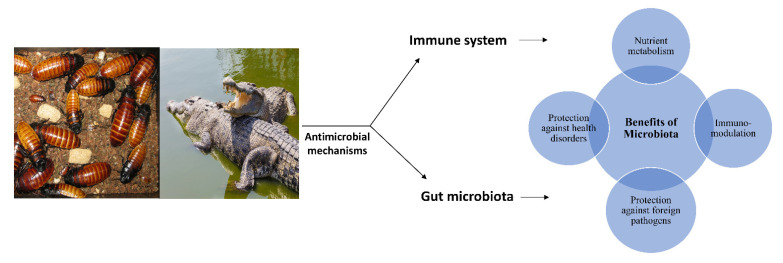
Gut bacteria of animals maybe a potential source of anti-infectives.

The antimicrobial protection of animals flourishing in their natural environments, which expose them to numerous microorganisms, may be due to (i) the immune system of these animals and/or (ii) the gut microbiome of these animals. The microbiome is indispensable for the functioning of its host, as it provides various benefits comprising nutrient metabolism, protection against pathogens, immunomodulation and potential protection against various health disorders. Thus, the gut bacteria of a number of animals who thrive in microorganism-rich environments, such as cockroaches, or whose diet involves carcasses, such as water monitor lizards, or those residing in environments laden with heavy metals and consuming rotten meat, such as crocodiles, may be a unique niche of novel bioactive molecules with potent efficacies against the multi-drug-resistant bacteria that typically result in high fatalities, as represented in Figure 1 [43,44,45]. Thus, this is a desirable topic of research that ought to be explored extensively.

Several previous studies have shown that the human gut microbiome plays a key role in the eradication of various pathogenic microorganisms that gain entry to the host by synthesizing antibiotics or molecules that modulate the immune response of the host, a phenomenon referred to as “immune-mediated colonization resistance” [46,47]. Thus, it is plausible that this is also the case in animals. This hypothesis is further supported by several reports that have assessed the potential antimicrobial activities of bacteria isolated from the gastrointestinal tract of various classes of animals, as summarized in Table 1.

### 2.1. Fish

Fish are thought to be among the most successful anamniotic ectotherms that reside in marine and freshwater habitats, and can adapt to live in some of the most extreme environments, such as those rich in hydrogen sulfide [59]. Although fish represent a vast taxonomic and ecologically diverse category, the comprehension of their gut microflora is only beginning to come to light, revealing low phylogenetic diversity, with *Proteobacteria*, *Firmicutes,* and *Bacteroidetes* representing approximately 90% of the fish intestinal microbiota. [60] Previously, a study investigated the antibacterial effects of the strains using antibacterial disc diffusion assays; *Bacillus* sp. PRV3 and *Bacillus* sp. PRV23, isolated from the gut of fish in Kerala, India; *Oreochromis mossambicus* (tilapia), *Hypselo barbuskolus* (koora), and *Punitus melanampyx* (kudukonda), and the herbivorous fish; *Nemacheilus menoni* (ayira) and *Channa murulius* (cherumeen). The results showed that fish bacterial metabolites were able to inhibit the activities of several pathogenic bacteria, namely *Klebsiella*, *S. aureus*, *Escherichia coli*, *Proteus mirabilis*, *Serratia marcescens*, *Vibrio parahaemolyticus*, and *Vibrio cholorae* [48]. Moreover, the gas chromatography–mass spectrometry results for the cell-free metabolite suspension revealed the presence of molecules with previously reported antimicrobial/antibacterial potentials, namely Neopentyl Glycol, Hentriacontane, Phenol, 2,4-Bis(1,1-Dimethylethyl, Heptacosane, and Methyl 3-(1-Pyrrolo)Thiophene-2, some of which were plant metabolites [49,61,62,63,64,65]. Of note, the study also reported that those bacteria inhabiting the gut of the fish produced secondary metabolites that reduced the viability of two cancer cells (HeLa and MCF-7), and furthermore, exhibited an apoptosis-like effect in cells post treatment [48].

Moreover, in our laboratory, the antibacterial efficacies of metabolites produced by bacteria (*E. coli*, G-pos-*bacilli*, *S. aureus*, *Staphylococcus auricularis*, *Aeromonas hydrophila*, and G-neg-*bacilli*) isolated from the gut of tilapia fish (*Oreochromis mossambicus*) was assessed against the clinical isolates: *Streptococcus pyogenes* ATCC 49399, *E. coli* K1 MTCC 710859, *P. aeruginosa* ATCC 10145, methicillin-resistant *S. aureus* MTCC 381,123, and *E. coli* K-12 MTCC 817,356 (non-clinical isolate) via bactericidal assays investigating bacterial viability in comparison to controls (the antibiotic gentamicin and Roswell Park Memorial Institute (RPMI) media). The results revealed that the gut bacteria of tilapia exhibited bactericidal efficacy against *P. aeruginosa* and *E. coli* K1, while only *S. aureus* did not exhibit bactericidal activity against MRSA, *S. auricularis* and *A. hydrophila*, and Gram-negative-*bacilli* exhibited bactericidal activity against *S. pyogenes* [49]. In another study, *Bacillus licheniformis* (P40 strain) isolated from the intestines of teleost fish *Leporinus* sp. synthesized an antibacterial peptide that exhibited antibacterial activity against *Bacillus cereus*, *L. monocytogenes*, and *Streptococcus* spp. [50].

Additionally, the lactic acid bacteria *Lactobacillus* sp. isolated from the gut of mullet fish (*Mugil cephalus*), were found to produce a 18kDa bacteriocin [52]. *Salmonella enterica* subsp. *enterica* serovar *Typhimurium* ATCC 14,028 and *Listeria monocytogenes* ATCC 19,115 isolated from beluga (*Huso huso*) and Persian sturgeon (*Acipenser persicus*) have been reported to produce 5 and 3 kDa bacteriocins that exhibit antibacterial activity against *A. hydrophila*, *E. coli*, *S. aureus*, *Vibrio anguillarum*, *Listeria* spp., *Salmonella* spp., and *B. cereus* [52]. In a study, the antibacterial potential of 27 bacteria isolated from the freshwater fish *Catla*, *Cyprinus carpio, Cirrhinus mirigala,* and *Labeo rohita* were assessed, and the results showed that select bacteria caused growth inhibition of *A. hydrophila* [53]. Another study identified the antibacterial activity of secondary metabolites produced by *Actinobacteria* isolated from the gut of two fish species, *Schizocypris altidorsalis* and *Schizothorax zarudnyi*. Some of the isolates exhibited antibacterial effects against *Streptomyces*, *Nocardiopsis*, *Micromonospora,* and *Saccharomonospora* species [54].

### 2.2. Reptiles

Extant non-avian reptiles are ectothermic amniotes and vertebrates that reside on every continent except Antarctica, and inhabit almost all biomes, including terrestrial, freshwater, and marine habitats, which may expose them to a variety of microorganisms, radiation, and/or heavy metals [31,37]. At present, there are limited studies on the gut microbiome composition of reptiles; however, with the availability of next-generation sequencing technologies, it has been revealed that the core gut microbiome of reptiles consists of *Proteobacteria*, *Firmicutes*, and *Bacteroidetes*, and that reptile gut bacterial communities are more comparable to those of birds than those of mammals [28]. Recently, the gut microbial compositions of four farmed snakes in China were elucidated [66]. The study revealed that the most abundant phyla were *Bacteroidetes*, *Proteobacteria*, *Firmicutes*, *Fusobacteria*, and *Actinobacteria*. The authors hypothesized that the host species is a significant influence affecting gut microbiome diversity, and affirmed the need to investigate whether the immunity and growth of farmed snake populations may be augmented by inoculating fecal suspensions from healthy wild snakes [66]. Another study revealed the structure and distribution of gut bacteria in various parts of the gastrointestinal tract of the snake species *Rhabdophis subminiatus* [67]. Furthermore, a study examined 22 snakes of three different species from the Philippines and investigated whether the host ecology and species differences were correlated with differences in microbial diversity within the gut and mouth [68]. These three species reside in three varying habitats: marine, semi-arboreal, and arboreal. The data obtained were indicative that the microbial diversity of the gut microbiome was correlated with host ecological and phylogenetic differences [68].

Studies investigating the effects of gut microbial metabolites, however, are few and far between. Recently, studies were conducted to examine the efficacy of bacterial metabolites produced from the gut microbiota of the python, water monitor lizard, and turtle [49]. Antibacterial activities of metabolites produced by the gut bacteria (*Citrobacter freundii*, *Bacillus paramycoides*, *Citrobacter braakii*, *Bacillus albus*, *P. mirabilis*, and *Escherichia fergusonii*) isolated from the gut of python (*Malayopython reticulatus*) were assessed using bactericidal assays. The results revealed that the metabolites from *C. freundii*, *C. braakii*, *P. mirabilis*, and *E. fergusonii* exhibited potent antibacterial activity against MRSA, while the metabolites from all bacteria except *E. fergusonii* exhibited antibacterial effects against *S. pyogenes* and *P. aeruginosa* [49]. Later, bacteria (*Enterobacter cloacae*, *A. hydrophila*, and *P. aeruginosa*) cultivated from the gut of *Cuora amboinensis* (turtle) were subjected to conditioned media (CM) preparation, which is a cell-free bacterial metabolite suspension. The overall results from the study demonstrated that the CM from the isolated bacteria exhibited antibacterial effects against various Gram-positive (*B. cereus*, methicillin-resistant *S. aureus* and *S. pyogenes*) and Gram-negative (*Klebsiella pneumoniae*, *S. marcescens*, *P. aeruginosa*, *Salmonella enterica*, and neuropathogenic *E. coli* K1) pathogenic bacteria [49].

In another very important study, a plethora of bacteria—*C. freundii*, *A. hydrophila*, *E. coli*, *S. aureus*, *P. mirabilia,* and *Staphylococcus* sp.—were isolated from the gut of a water monitor lizard (*Varanus salvator*) and were subjected to CM preparation [56]. The CM were tested for their antibacterial efficacy using bactericidal assays, and results revealed that CM from *P. mirabilis*, *E. coli*, *Staphylococcus* sp., and *S. aureus* exhibited antibacterial activity against *B. cereus*, while CM from *C. freundii*, *A. hydrophila*, *E. coli*, and *Staphylococcus* sp. exhibited antibacterial activity against MRSA. CM from *E. coli*, *C. freundii*, *Staphylococcus* sp., and *S. aureus* revealed antibacterial efficacy against *S. pyogenes,* and CM from *Proteus mirabilis, C. freundii*, *E. coli*, *Staphylococcus* sp., and *S. aureus* exhibited antibacterial activity against *P. aeruginosa*. Finally, CM from *P. mirabilis*, *A. hydrophila*, *C. freundii*, *E. coli*, and *Staphylococcus* sp. exhibited antibacterial activity against *S. enterica*, and all CM exhibited antibacterial activity against *K. pneumoniae* and *S. marcescens* [56]. Furthermore, liquid chromatography–mass spectrometry (LC–MS) results revealed the presence of secondary metabolites with previously reported antibacterial activity alkaloids, flavonoids, terpenes, hydroxylated fatty acids, oxygenated fatty acids, and pyrazine derivative. The presence of Dehydrocurdione, a molecule responsible for the antibacterial effect of turmeric, was also noted [56,69].

Interestingly, in previous studies it was also shown that CM prepared from the gut bacteria of the crocodile (*Crocodylus porosus*), particularly *Aeromonas dhakensis*, *Pseudomonas guezennei,* and *P. aeruginosa,* exhibited potent effects against breast, cervical, and prostate cancer cell lines. LC–MS results further supported these findings, as this revealed the presence of molecules with reported anticancer activity in the active CM, namely PD 98,059 and L, L-Cyclo(leucylprolyl). Moreover, LC–MS revealed molecules with previously reported antibacterial activity in the active CM: lactic acid, F-Honaucin A, L, L-Cyclo(leucylprolyl), Granisetron metabolite 1, and Phenylethylamine, suggesting that those CM might also exhibit antibacterial potential [70]. Additionally, it was reported that lactic acid, F-Honaucin A, L, L-Cyclo(leucylprolyl), Granisetron metabolite 1, and Phenylethylamine exhibited anticancer activity against breast, cervical, and prostate cancer cell lines. Again, this was supported by LC–MS analysis, which showed molecules with reported anticancer activity in the active CM, namely C75, 3-Butylidene-7-hydroxyphthalide, Estrone 16-oxime, Enigmol, Proglumide, and S-Allyl-L-cysteine, and molecules with reported antibacterial activity, namely Benzocaine and Quindoxin [70]. However, the antibacterial effects of these CM need to be investigated in future studies.

### 2.3. Birds

Avian reptiles/birds are a diverse group of amniotic endothermic vertebrates with a global distribution, and many species undergo lengthy seasonal migrations across great distances [71,72]. Furthermore, a variety of diets and life history strategies are utilized by birds, and thus elucidating their microbiome is of interest [71,72]. The gut microbiome of birds is dominated by members of the *Firmicutes*, with *Actinobacteria*, *Bacteroidetes*, and *Proteobacteria*, although the relative proportions of these is known to vary amongst different species [72].

Compared to other nonmammalian vertebrates, comprehension of the bird microbiome is greater; however, most avian microbiome reports have been focused on economically important species; for example, chicken and turkey [72]. In a study, the antimicrobial activity of cell-free supernatant (CFS) from *Enterococcus faecium* KQ 2.6 isolated from the fecal matter of *Pavo cristatus* (peacock) was assessed against a plethora of pathogenic bacteria: *Bacillus subtilis*, *B. cereus*, *S. pyogenes*, *S. aureus*, *E. faecalis*, *E. coli*, *P. aeruginosa*, *K. pneumoniae*, *Salmonella enterica* sub. *enterica* serovar *paratyphi, Staphylococcus epidermidis*, *Aspergillus niger,* and *Candida albicans*. The results generated showed that the CFS prepared exhibited antibacterial activity against the selected bacteria [57]. In our laboratory, the antibacterial potential of CM prepared from *Escherichia fergusonii*, *Shigella flexneri*, *B. cereus*, and *E. faecalis* isolated from the gastrointestinal tract of a wild *Gallus gallus domesticus* (Chicken) was assessed and the results indicated that all CM exhibited bactericidal efficacies against *E. coli* K1, *S. pyogenes,* and *P. aeruginosa*, while all CM except *S. flexneri* exhibited bactericidal effects against MRSA [49]. Moreover, we previously reported that *B. cereus* and *Bacillus velezensis* isolated from the fecal matter and gut of *Columba livia domestica* (pigeon) exhibited anticancer activity against cervical, breast, and prostate cancer cell lines, and exhibited cytotoxicity towards HeLa cervical cancer cells at IC_50_ concentration of 10.65 and 15.19 µg/mL. LC–MS results for the CM of these active bacteria showed the presence of molecules with reported anticancer (dihydroxymelphalan) and antibacterial activity (citric acid) [73].

### 2.4. Amphibians

Amphibians are ectothermic, tetrapod vertebrates that reside in a variety of habitats, living in terrestrial, arboreal, fossorial, or freshwater aquatic environments, and are amongst the world’s most vulnerable groups of animals, with 40% of these species in danger of extinction [74,75]. There is limited information regarding the gut microbiome of amphibians, and recently, a study was conducted that investigated the correlation between the diversity of diet and the gut microbiome of adult fire salamanders in Belgian forests, using high-throughput DNA metabarcoding [75]. It was shown that the diet composition was driven by sex, and this influenced the microbiome composition in the fire salamander. However, no correlation was observed between diet diversity and gut microbiome diversity. Another study revealed that the leopard frog gut bacterial communities underwent significant changes when going through metamorphosis [76].

Recently, the antibacterial properties of two bacteria, namely *P. mirabilis* and *Proteus vulgaris*, isolated from the American bull frog gut (*Lithobates catesbeianus*) were elucidated against several clinical isolates, namely *E. coli* K1 MTCC 710859, *P. aeruginosa* ATCC 10145, methicillin-resistant *S. aureus* MTCC 381123, *S. pyogenes* ATCC 49,399, and the non-clinical isolate of *E. coli* K-12 MTCC 817,356 [49]. Antibacterial activity was established via bactericidal assays conducted following the preparation of the metabolites from the two bacterial species. The results revealed that metabolites from both bacteria exhibited antibacterial effects against *P. aeruginosa, E. coli* K1, and *S. pyogenes*, while only *P. vulgaris* exhibited antibacterial activity against MSRA. Moreover, post heat inactivation, the metabolite suspension prepared from each bacterium retained their antibacterial activity, hinting that the active molecule(s) responsible for the antibacterial activity might not be proteinaceous in nature [49]. Thus, further studies must be conducted focusing on amphibian gut microbial metabolites as well as gut microbiome composition, with the aim of unveiling molecules with antibacterial potential to alleviate the burden of infectious diseases among humans, as well as for the conservation and health management of amphibians [77].

### 2.5. Invertebrates

Invertebrates are thought to constitute the majority of animal species, comprising an estimated 97% of all species on earth, and thus residing in a variety of ecosystems, such as deep in the ocean and in surface water, soil, and other terrestrial areas, including those with the most difficult conditions for biological life. Furthermore, their diet consists of a variety of food [78,79,80]. Although previously neglected by scientists, currently the most studied model organisms are invertebrates, namely *Drosophila melanogaster* and *Caenorhabditis elegans* [80]. Recently, studies have begun to explore the impact of the gut and its effects on ageing, using invertebrates as model organisms [80]. Despite the distinct differences between the invertebrate gut and the corresponding microbiome of mammalian models and humans, several comparisons in regard to gut dysbiosis, immune function, and intestinal decline can be made; thus, utilizing the gut microbiome of these abundant species for the benefit of human and animal health is warranted, given the key role these species play in the food web, as well as in organic matter decomposition [80].

In a recent study, the antibacterial potential of CM produced from the gut bacteria of the mud crab (*Scylla serrata*), red-headed centipede (*Scolopendra subspinipes*) and rose hair tarantula (*Grammostola rosea*) was assessed [49]. *Kluyvera georgiana*, *Lysinibacillus fusiformis*, *P. aeruginosa*, and *Bacillus proteolyticus* were isolated from the centipede, while *S. aureus*, *B. subtilis*, *Pseudomonas putida*, and G-neg-*bacilli* were isolated from the tarantula, and *Proteus alimentorum*, *K. pneumoniae*, and *P. vulgaris* isolated from the crab. The results showed that all CM from the centipede exhibited antibacterial activity against *E. coli* K1 and *P. aeruginosa*, apart from *B. proteolyticus*. Additionally, all CM exhibited antibacterial activity against *S. pyogenes* and MRSA. From the tarantula, all CM exhibited antibacterial activities against *E. coli* K1 and *P. aeruginosa*, besides the G-neg-*bacilli*; all CM exhibited antibacterial activity against *S. pyogenes,* and only CM from *P. putida* and G-neg-*bacilli* exhibited antibacterial efficacy against MRSA. From the crab, *K. pneumoniae* and *P. vulgaris* exhibited bactericidal efficacy against *S. pyogenes*, *E. coli* K1, and *P. aeruginosa*, while none of the CM exhibited antibacterial effects against MRSA [49]. Another interesting study revealed that the larvae of *Lucilia sericata* or sheep blowfly, which are utilized in maggot debridement therapy, had potent antimicrobial effects [81].

Cockroaches are known to share ecological niches with humans and other animals, plausibly being exposed routinely to a variety of microorganisms in their habitats, and thus are of interest [32,33,82]. Previous studies revealed the potent antibacterial effects of extracts from various body organs of cockroaches (*Periplaneta americana*) against MRSA and neuropathogenic *E. coli* K1 [82]. More recently, the antibacterial potential of CM prepared from gut bacteria isolated from two species of cockroach was assessed. *S. marcescens* and *E. coli* were isolated from *Gromphadorhina portentosa* (Madagascar), while *Klebsiella* sp., *Citrobacter* sp., *Bacillus* sp., *Klebsiella* sp., and *Streptococcus* sp. were isolated from *Blaptica dubia* (Dubia) cockroach, and metabolite suspensions were prepared before elucidating the bactericidal properties [58]. The results showed that all CM exhibited antibacterial activity against *B. cereus*, while only CM from *S. marcescens, E. coli, Klebsiella* sp., and *Citrobacter* sp. exhibited antibacterial activity against MRSA, and all CM except *Bacillus* sp. exhibited antibacterial activity against *S. pyogenes* [58]. Moreover, the study revealed that CM from those cockroach species also exhibited anti-amoebic efficacy [58]. In another report, *P. aeruginosa* and *B. subtilis* isolated from the gut of *Heterometrus spinifer* (scorpion) depicted anticancer activity against cervical, breast, and prostate cancer cell lines. Through LC–MS, we also noted that those bacteria produced molecules with previously reported anticancer activity; 3-butylidene-7-hydroxyphthalide, U-0126, and proglumide. However, the LC–MS revealed that the active bacteria also produce molecules with reported antibacterial activities, namely dextromethorphan, citric acid, and 3-Butylidene-7-hydroxyphthalide [83].

## 3. Conclusions

Previously, antimicrobials were sourced from soil bacteria, and many major antibiotics and antifungals were elucidated [84,85]. Due to the overuse and decline in the development of antimicrobials, there is an urgent need to discover novel molecules [85]. Recent studies have depicted that various species possess molecules with antibacterial potential. In this regard, the gut microbiome of fish, reptiles, birds, amphibians, and invertebrates may produce molecules that exhibit antibacterial activity against other bacterial strains, but their precise identity and mode of action is not yet known, and mechanistic studies are warranted. This will require synthesizing bioactive molecules from the gut microbiome of animals, target identification and screening, testing of the purified molecule, lead optimization, suggested modification of identified molecules making them specific to target site, evaluation in the clinical setting comprising both pre-clinical and clinical studies, product analysis, and approval.

With recent advances in next-generation sequencing platforms, such as proteomics, transcriptomics, and metabolomics, the genomic and epigenomic aspects of these species need to be investigated. In this regard, future studies should focus on anaerobic bacteria and other unculturable bacteria/other microbes that may also be a potential source of novel molecules. Consequently, these microorganisms should also be recovered and the activities of their metabolites should be determined. Recent analyses of crocodilian genomes showed that these have evolved very slowly over the past several million years, and it would be useful to understand how these slow-evolving species are able to thrive over millions of years, despite ongoing environmental changes [86]. Alternatively or concurrently, the development of these gut microbial species and/or metabolites for use as probiotics should be explored. For example, in 1917, a German corporal seemed to be immune to dysentery during an ongoing epidemic. It was postulated that the flora of this soldier contained an *E. coli* strain that exerted antagonistic activities against various pathogens. This specific strain of *E. coli* was cultivated and patented as the therapy “Mutaflor©”, and is widely available in Germany and other countries to the present day [87]. Thus, expedited studies involving the implantation of selected gut microbiome species or their metabolites from animals, such as crocodiles, cockroaches, or snakes, into mammalian models of infectious diseases or aging are encouraged, with a view to the potential future development of probiotics that may be beneficial for human and animal health. Moreover, as insects such as cockroaches possess an innate system and rely on this entirely instead of adaptive immunity, it is imperative to study these mechanisms as well as to examine the various antimicrobial peptides produced by these interesting species [88]. Nonetheless, it is also important to highlight that there are gaps in the understanding of the interaction between the microbiome and the host and the pathway of its metabolites, as well as how these metabolites may influence the microenvironment, and further mechanistic studies should be accomplished, as previously described [89].

## 4. Significance and Impact

The majority of antibiotics currently available in the market were developed from bacteria isolated from the soil, based on the concept of microbial competition. However, this low-hanging fruit has already been picked; hence, there is a need to mine bacteria from unusual and novel sources. With this in mind, it is important to note that animals such as cockroaches, snakes, crocodiles, and water monitor lizards come across microorganisms regularly, yet flourish in these environments. These species must have developed methods to defend themselves to counter these pathogens. Besides their immunity, the gut microbiome of these animals/pests may offer a potential source of novel antimicrobials and should be the focus of prospective research.

## Figures and Tables

**Table 1:** Selected gut bacteria of reported animals and their antibacterial properties.

Animal		Bacteria	Efficacy against Test Organisms	Molecules with Previously Reported Antibacterial Activity
Class	Scientific Name			
Fish	*Hypselo barbuskolus* (koora), *Oreochromis mossambicus* (tilapia), *Punitus melanampyx* (kudukonda), *Channa murulius* (cherumeen) & *Nemacheilus menoni* (ayira)	*Bacillus* sp. PRV3 & *Bacillus* sp. PRV23	*Escherichia coli*, *Klebshiella*, *Proteus mirabilis*, *Serratia marcescens*, *Staphylococcus aureus*, *Vibrio parahaemolyticus*, & *Vibrio chlorae* [48,49].	Neopentyl Glycol, Hentriacontane, Phenol, 2,4-Bis(1,1-Dimethylethyl, Heptacosane & Methyl 3-(1-Pyrrolo)Thiophene-2 [48,49].
	*Oreochromis mossambicus* (tilapia)	*Escherichia coli*, *Staphylococcus auricularis*, G-pos-*bacilli*, *Staphylococcus aureus*, *Aeromonas hydrophila*, & G-neg-*bacilli*	*Escherichia coli* K1, *Pseudomonas aeruginosa*, methicillin-resistant *S. aureus*, *Streptococcus pyogenes* & *Escherichia coli* K-12 [50].	
	*Leporinus* sp.	*Bacillus licheniformis* strain P40	*B. cereus*, *L. monocytogenes*, & *Streptococcus* spp. [51].	
	*Mugil cephalus* (mullet fish)	*Lactobacillus* sp. lactic acid bacteria (LBA)		18kDa bacteriocin (further characterization is needed) [52].
	*Huso* (beluga) & *Acipenser persicus* (persian sturgeon)	*Listeria monocytogenes* & *Salmonella enterica* subsp. *enterica* serovar *Typhimurium*	*Escherichia coli*, *Listeria* spp., *Salmonella* spp., *Staphylococcus aureus*, *Aeromonas hydrophila*, *Vibrio anguillarum*, & *Bacillus cereus* [52].	5 & 3 kDa bacteriocins [52].
	*Catla catla*, *Labeo rohita*, *Cirrhinus mirigala* & *Cyprinus carpio*		*Aeromonas hydrophila* [53].	
	*Schizothorax zarudnyi* & *Schizocypris altidorsalis*	*Actinobacteria*	*Streptomyces*, *Nocardiopsis*, *Micromonospora* & *Saccharomonospora* species [54].	
Reptile	*Malayopython reticulatus* (python)	*Citrobacter freundii*, *Citrobacter braakii*, *Proteus mirabilis*, & *Escherichia fergusonii*	*Pseudomonas aeruginosa*, methicillin-resistant *S. aureus*, *Streptococcus pyogenes* [49].	
	*Cuora amboinensis* (turtle)	*Enterobacter cloacae*, *Aeromonas hydrophila*, & *Pseudomonas aeruginosa*	*Bacillus cereus*, *Streptococcus pyogenes*, methicillin-resistant *Staphylococcus aureus*, *Escherichia coli* K1, *Serratia marcescens*, *Pseudomonas aeruginosa*, *Salmonella enterica* & *Klebsiella pneumoniae* [55].	
	*Varanus salvator* (water monitor lizard)	*P. mirabilis*, *A. hydrophila*, *C. freundii*, *E. coli*, *Staphylococcus* sp. & *S. aureus*	*Bacillus cereus*, *methicillin-resistant Staphylococcus aureus*, *Pseudomonas aeruginosa*, *Streptococcus pyogenes*, *Serratia marcescens*, & *Klebsiella pneumoniae* [56].	Flavonoids, alkaloids, terpenes, oxygenated fatty acids, hydroxylated fatty acids, & pyrazine derivative [56].
Bird	*Pavo cristatus* (peacock)	*Enterococcus faecium* KQ 2.6	*Bacillus subtilis*, *B. cereus*, *S. pyogenes*, *S. aureus*, *Staphylococcus epidermidis*, *E. faecalis*, *E. coli*, *P. aeruginosa*, *K. pneumoniae*, *Salmonella paratyph*i, *Candida albicans* & *Aspergillus niger* [57].	
	*Gallus gallus domesticus* (chicken)	*Escherichia fergusonii*, *Shigella flexneri*, *B. cereus*, & *E. faecalis*	*E. coli* K1, *S. pyogenes*, *P. aeruginosa* & methicillin-resistant *Staphylococcus aureus* [49].	
Amphibian	*Lithobates catesbeianus* (American bull frog)	*Proteus mirabilis* & *Proteus vulgaris*	*E. coli* K1, *P. aeruginosa*, *S. pyogenes* & methicillin-resistant *Staphylococcus aureus* [49].	
Invertebrate	*Scolopendra subspinipes* (red-headed centipede)	*Lysinibacillus fusiformis*, *Kluyvera georgiana*, *P. aeruginosa*, & *Bacillus proteolyticus*	*E. coli* K1, *P. aeruginosa*, *S. pyogenes* & methicillin-resistant *Staphylococcus aureus* [50].	
	*Grammostola rosea* (rose hair tarantula)	*S. aureus*, *B. subtilis*, *Pseudomonas putida*, & G-neg-*bacilli*	*E. coli* K1, *P. aeruginosa*, *S. pyogenes* & methicillin-resistant *Staphylococcus aureus* [50].	
	Scylla serrata (mud crab)	*K. pneumoniae*, *Proteus alimentorum*, & *P. vulgaris*	*E. coli* K1, *P. aeruginosa*, *S. pyogenes* & methicillin-resistant *Staphylococcus aureus* [50].	
	Gromphadorhina portentosa (Madagascar cockroach)	*S. marcescens* & *E. coli*	*B. cereus*, *S. pyogenes* & methicillin-resistant *Staphylococcus aureus* [58].	
	*Blaptica dubia* (Dubia cockroach)	*Klebsiella* sp., *Citrobacter* sp., *Bacillus* sp., *Klebsiella* sp., & *Streptococcus* sp.	*B. cereus*, *S. pyogenes* & methicillin-resistant *Staphylococcus aureus* [58].	

## Data Availability

Data sharing is not applicable for this article, as no datasets were generated or analyzed during the current study.
